# Homogeneously Blending PBAT with Silanized Cellulose for Composite Film: Characterization and Physicochemical Property

**DOI:** 10.3390/polym18070875

**Published:** 2026-04-02

**Authors:** Ce Zhao, Xinxin Yan, Zhou Zhou, Lukuan Guo, Shilong Yang, Zhen Chen, Fengwei Jia, Junlong Song, Jiaqi Guo

**Affiliations:** 1 State Key Laboratory for Development and Utilization of Forest Food Resources and Jiangsu Provincial Key Lab of Sustainable Pulp and Paper Technology and Biomass Materials, Nanjing Forestry University, Nanjing 210037, China; 19166486198@njfu.edu.cn (C.Z.); 19852609685@163.com (Z.Z.); glk8362@163.com (L.G.); junlong.song@gmail.com (J.S.); 2 Advanced Analysis and Testing Center, Nanjing Forestry University, Nanjing 210037, China; yshl6072@njfu.edu.cn; 3 State Key Laboratory for Development and Utilization of Forest Food Resources, Jiangsu Co-Innovation Center of Efficient Processing and Utilization of Forest Resources, Nanjing Forestry University, Nanjing 210037, China; chenzhen1719@njfu.edu.cn; 4 Department of Nuclear Medicine, The First Affiliated Hospital with Nanjing Medical University, Nanjing 210029, China; 5 Shandong Henglian New Materials Co., Ltd., Weifang 261106, China; jiafengwei.love@163.com

**Keywords:** PBAT, silanized cellulose, crystallization behavior, mechanical properties, hydrophobicity

## Abstract

Improving the interfacial compatibility between cellulose and poly(butylene adipate-co-terephthalate) (PBAT) is critical for enhancing the performance of PBAT-based composites. Here, microcrystalline cellulose (MCC) was homogeneously silanized at the molecular chain level using *t*-hexyldimethylchlorosilane (TDMS-Cl) as the modifier, yielding *t*-hexyldimethylsilylated cellulose (TDMS-Cell). TDMS-Cell/PBAT composite films were then prepared by solution blending and casting in tetrahydrofuran (THF). Structural characterizations confirmed the successful grafting of TDMS-Cl onto cellulose chains, resulting in TDMS-Cell with a degree of substitution of approximately 2. Microstructural observations combined with thermal analysis revealed that TDMS-Cell exerted a dual effect on the crystallization behavior of PBAT: it acted as a heterogeneous nucleating agent that increased the crystallization temperature, while the pronounced steric hindrance simultaneously suppressed crystal growth. Mechanical testing showed that simultaneous strengthening and toughening were achieved at an optimal TDMS-Cell loading of 3–5 wt%. Specifically, the tensile strength increased from ~16 MPa for neat PBAT to 21 MPa (31.25% improvement), and the elongation at break increased from ~700% to 964% (37.7% improvement). In addition, the incorporation of an appropriate amount of TDMS-Cell effectively enhanced the surface hydrophobicity of the composite films. At higher filler loading, however, solvent evaporation-induced phase separation led to self-aggregation of TDMS-Cell, which in turn deteriorated both the mechanical properties and surface hydrophobicity of the composites. Overall, this work systematically elucidates the structure–property relationships of silanized cellulose/PBAT composites in a homogeneous solution system, providing a rational basis for interfacial design and property optimization of PBAT/biomass-based composite materials. The prepared TDMS-Cell/PBAT composite films with balanced mechanical strength, tunable crystallization behavior, and improved surface hydrophobicity exhibit great potential for practical applications in high-performance flexible packaging materials, functional film substrates, lightweight composite structural components, and tunable hydrophobicity coating substrates.

## 1. Introduction

Polymer materials have become indispensable in modern industry and daily life owing to their excellent processability and cost effectiveness [1]. However, with the depletion of petrochemical resources and the increasing environmental burden caused by plastic waste, the development of renewable and environmentally friendly biodegradable polymers has become an important research direction in materials science [2,3]. Poly(butylene adipate-co-terephthalate) (PBAT) is a representative biodegradable aliphatic-aromatic copolyester that combines good mechanical toughness with favorable processability and can gradually degrade in soil or compost environments. Therefore, it is considered a promising alternative to conventional polyolefin packaging materials [4].

Although PBAT exhibits relatively balanced overall performance among polymers, its rigidity and strength still do not meet the requirements of certain applications, and a reinforcing phase is therefore commonly introduced to tailor its properties. Under the premise of maintaining environmental friendliness, biomass-based materials such as cellulose and lignin have attracted extensive attention because of their wide availability and excellent mechanical properties [5]. Among them, cellulose is characterized by high crystallinity and high specific strength and is regarded as an ideal reinforcing phase for the composite modification of polymers [6]. However, cellulose also suffers from a notable drawback: its surface is rich in hydrophilic hydroxyl groups [7], which not only leads to strong hydrophilicity but also promotes self-aggregation through intermolecular hydrogen bonding [8]. This, in turn, makes it difficult to achieve uniform dispersion of cellulose in the hydrophobic or weakly polar PBAT matrix, and insufficient interfacial compatibility often becomes a key factor limiting further improvement in composite performance [9]. To enhance the interfacial interaction between cellulose and the polymer matrix and thereby improve the overall properties of the composites, previous studies have commonly adopted modification strategies such as esterification and amidation [10,11]. These approaches regulate surface chemical properties by grafting functional groups, thus improving the dispersibility and interfacial adhesion of cellulose in polyester systems such as PBAT. Among these strategies, silylation is considered an effective route to enhance compatibility. For example, Wang et al. modified cellulose nanofibers with 3-aminopropyltriethoxysilane and incorporated them into a PBAT matrix via melt processing [12], while Dhali et al. surface-grafted cellulose nanocrystals with vinyl silane and prepared the corresponding composites by solution casting, confirming the positive effect of silylation on interfacial compatibility [13]. These studies demonstrate that silylation can improve the compatibility and interfacial interactions between cellulose and PBAT to a certain extent.

Most existing studies have combined and applied cellulose modified by surface modification with PBAT, while few studies have focused on the combination of homogeneously modified cellulose and PBAT [14]. Such approaches usually require the use of small-sized cellulose to facilitate dispersion and modification, which not only imposes stringent requirements on the raw materials but also involves relatively cumbersome preparation processes. In contrast, homogeneous silanization of cellulose at the molecular chain level can fundamentally alter its intermolecular interactions and solubility, thereby leading to dispersion states and interfacial interactions in polymer matrices that differ from those obtained by conventional surface modification. Previous studies by Hirose [15], Koschella [16], and co-workers have demonstrated the synthesis of various silylated celluloses via homogeneous modification. However, systematic investigations into the structure–property relationships of composite systems composed of homogeneously modified cellulose and PBAT are still lacking.

To address these issues, microcrystalline cellulose (MCC) was dissolved in a *N*,*N*-dimethylacetamide/lithium chloride (DMA/LiCl) system and subjected to homogeneous silanization using *t*-hexyldimethylchlorosilane (TDMS-Cl) as the modifier, yielding homogeneously modified *t*-hexyldimethylsilylated cellulose (TDMS-Cell). This modification endowed TDMS-C with a polarity similar to that of PBAT and rendered it soluble in organic solvents such as tetrahydrofuran (THF), chloroform (CHCl_3_), and 1,2-dichloroethane (C_2_H_4_Cl_2_), which facilitates improved dispersion in the PBAT matrix and enhanced interfacial interactions. Subsequently, TDMS-Cell/PBAT composite films were fabricated by homogeneous solution blending. The effects of TDMS-Cell loading on the mechanical properties, surface hydrophobicity, crystallization behavior, and thermal stability of the composite films were systematically investigated. This work supplements the understanding of the structure–property relationships of silanized cellulose/PBAT composites prepared in a homogeneous solution system and clarifies the regulation mechanisms governing composite properties, thereby providing theoretical and technical references for the development and application of related biodegradable composites.

## 2. Materials and Methods

### 2.1. Materials

PBAT pellets (number-average molecular weight(M_n_) ≈ 120,000), chlorodimethylsilane (purity 95%), THF (analytical grade), imidazole (purity 99%), and N,N-dimethylacetamide (DMA, purity ≥ 99.8%) were purchased from Shanghai Macklin Biochemical Technology Co., Ltd. (Shanghai, China). 2,3-Dimethyl-2-butene (purity ≥ 98%) and anhydrous aluminum trichloride (analytical grade) were obtained from Shanghai Aladdin Biochemical Technology Co., Ltd. (Shanghai, China). Microcrystalline cellulose (MCC powder, chemical pure) was supplied by Sinopharm Chemical Reagent Co., Ltd. (Shanghai, China). All reagents were used as received without further purification.

### 2.2. Synthesis of TDMS-Cl

The synthesis of TDMS-Cl was carried out with slight modifications based on the work reported by Savela et al. [17]. Amounts of 10 mL of 2,3-dimethyl-2-butene and 8 mL of chlorodimethylsilane were mixed, followed by the addition of 0.6 g of anhydrous aluminum trichloride as a catalyst; the mixture was stirred at room temperature (RT) for 4 h to ensure sufficient reaction. After completion of the reaction, anhydrous aluminum trichloride was removed by filtration, and unreacted raw materials were distilled off at 100 °C. The final colorless and transparent oily liquid was the target product.

### 2.3. Synthesis of TDMS-Cell

TDMS-Cell was synthesized according to the method reported by Koschella et al. [18]. Briefly, 1 g of MCC was added to 25 mL of N,N-dimethylacetamide, and the mixture was stirred in an oil bath at 120 °C for 2 h to fully swell the MCC. After removal of the oil bath, 1.5 g of anhydrous lithium chloride was added, and the system was stirred and allowed to cool naturally to room temperature until the MCC was completely dissolved, as indicated by the formation of a transparent solution. Subsequently, 2 g of imidazole was added, and the mixture was stirred in an oil bath at 100 °C. After the imidazole was fully dissolved, 5 mL of TDMS-Cl was added over 5 min, and the reaction was allowed to proceed at 100 °C under continuous stirring. After 24 h, 20 mL of ethanol was added to precipitate the product completely. The precipitate was collected and washed repeatedly with ethanol and distilled water, affording a white powder, which was then dried under vacuum at 60 °C for 24 h to obtain crude TDMS-Cell. The crude product was further dissolved in tetrahydrofuran, centrifuged to remove impurities, and subsequently subjected to reprecipitation and drying to finally obtain high-purity TDMS-Cell powder.

### 2.4. Preparation of TDMS-Cell/PBAT Composite Films

**Figure 1:**
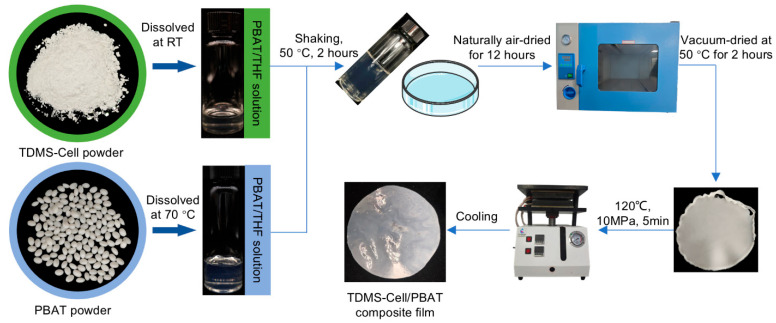
Preparation process of TDMS-Cell/PBAT composite films.

In this study, composite films were prepared via solution blending and casting (Figure 1). The detailed preparation procedure is as follows: 2 g of PBAT was dissolved in 10 mL of THF at 70 °C; TDMS-Cell powder was then dissolved in 5 mL of THF at room temperature with mass fractions of 1%, 3%, and 5% relative to PBAT (Table 1). The two solutions were mixed and shaken at 50 °C for 2 h to achieve uniform dispersion of PBAT and TDMS-Cell. After shaking, the mixture was poured into a glass Petri dish and placed in a fume hood to stand until THF was completely evaporated to form a film. The resulting film was further dried in a vacuum oven at 50 °C for 2 h to completely remove residual solvent. Finally, the film was hot-pressed using a flat vulcanizer at 120 °C and 10 MPa for 5 min. Uniform and dense TDMS-Cell/PBAT composite films with a thickness of 0.4 ± 0.1 mm were obtained and designated as P-C1%, P-C3%, and P-C5% according to the mass fractions of 1%, 3%, and 5%, respectively. Notably, to better characterize the effects of TDMS-Cell loading on the mechanical properties, hydrophobicity, and other key performances of the composites, additional groups (P-C10%, P-C20%, and P-C30%) were incorporated into this section, with TDMS-Cell added at mass fractions of 10%, 20%, and 30%, respectively.

### 2.5. Characterization and Methods

Fourier transform infrared spectroscopy (FTIR) measurements were performed using a Vertex 80v spectrometer (Bruker, Ettlingen, Germany) at a resolution of 2 cm^−1^ and a spectral range of 4000–400 cm^−1^.

Nuclear magnetic resonance (NMR) tests were carried out on an AVANCE III HD 600 MHz NMR spectrometer (Bruker, Ettlingen, Germany). TDMS-Cell was dissolved in deuterated chloroform for testing, and the degree of substitution (DS) was calculated from the characteristic peaks of the ^1^H NMR spectrum according to Equation (1) [19,20].

(1) $$DS=\frac{10A}{12B+A}$$

A: Integral area of methyl protons not directly bonded to Si in TDMS groups;
B: Integral area of protons on the glucosyl units of cellulose.

X-ray photoelectron spectroscopy (XPS) tests were conducted using an AXIS Ultra DLD X-ray photoelectron spectrometer (Shimadzu, Kyoto, Japan) with monochromatic Al Kα radiation (excitation energy = 1486.6 eV). The binding energy was calibrated using the C 1s characteristic peak at 284.8 eV as a reference. Combined with the atomic ratio of carbon to silicon, the degree of substitution (DS) can be calculated using Equation (2).

(2) $$DS=\frac{6N}{1-8N}$$

N: atomic ratio of Si to C.

X-ray diffraction (XRD) patterns of the samples were obtained using an Ultima IV X-ray diffractometer (Rigaku, Kyoto, Japan) using Cu Kα radiation (λ = 1.5418 Å), at 40 kV and 20 mA. Data were collected in reflection mode at a scanning rate of 5°/min over a 2θ scanning range of 5–50°. The relative crystallinity (X_c_) of the composite films was calculated using Equation (3) [21].

(3) $$X_{c}=\frac{\sum A_{c}}{\sum A_{c}+\sum A_{a}}\times 100\%$$

∑Ac is the sum of the diffraction peak areas obtained by fitting the crystalline regions; ∑Aa is the sum of the scattering peak areas obtained by fitting the amorphous regions.

Scanning electron microscopy (SEM) was performed using a Regulus 8100 cold-field emission scanning electron microscope (Hitachi, Tokyo, Japan) at an accelerating voltage of 3 kV to observe the cross-sectional morphologies of Au-sputtered samples.

Tensile tests for mechanical properties were carried out on an AGS-X universal testing machine (Shimadzu, Tokyo, Japan) at a crosshead speed of 20 mm/min. The specimens were rectangular strips of 15 mm × 5 mm, and at least 5 replicate samples were tested for each group.

Morphological observations were performed using an MP41 optical microscope (Mingmei, Guangzhou, China) (OM). For crystallization studies, the MP41 microscope equipped with a polarizer was used as a polarizing optical microscope (POM). Composite film samples were placed on a hot/cold stage system, melted at 180 °C for 5 min to erase thermal history, then rapidly cooled to 110 °C, and held isothermally to monitor crystallization behavior.

Thermogravimetric analysis (TG) was performed using a TG 209 F1 Libra thermogravimetric analyzer (Netzsch, Selb, Germany) under a nitrogen atmosphere. The heating rate was 10 °C/min, the nitrogen flow rate was 20 mL/min, and the test temperature range was 50–700 °C. Derivative thermogravimetric (DTG) curves were obtained by differentiating the TG data [22].

Differential scanning calorimetry (DSC) was conducted using a DSC 214 Polyma differential scanning calorimeter (Netzsch, Selb, Germany) under nitrogen protection. The samples was heated to 180 °C at 10 °C/min; held for 5 min to eliminate thermal history; and then cooled to 30 °C at cooling rates of 5 °C/min and 10 °C/min, respectively, to complete the measurements.

Water contact angle measurements were carried out using a DSA100S contact angle analyzer (Krüss, Hamburg, Germany) with a droplet volume of 2 μL. Images were captured 5 s after the droplet was deposited on the sample surface, and the contact angle values were calculated based on the images.

## 3. Results and Discussion

### 3.1. Synthesis and Characterization of TDMS-Cell

**Figure 2:**
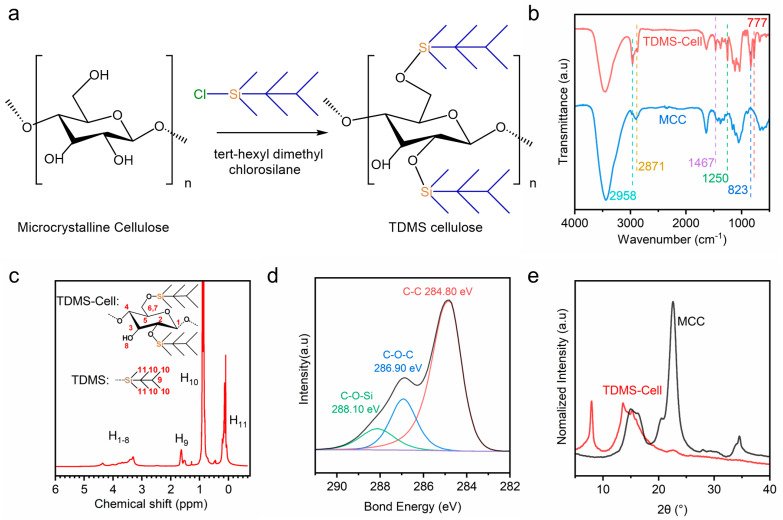
(**a**) Reaction mechanism of TDMS-Cl-modified MCC, (**b**) FTIR spectra, (**c**) ^1^H NMR spectra of TDMS-Cell, (**d**) high-resolution C 1s XPS spectrum of TDMS-Cell, (**e**) XRD spectra.

TDMS-Cell was synthesized by dissolving microcrystalline cellulose in an N,N-dimethylacetamide/lithium chloride system, followed by a nucleophilic substitution reaction with TDMS-Cl (using imidazole as the catalyst) to graft silyl groups. Figure 2 presents the reaction scheme and the structural characterization results for the synthesis of TDMS-Cell using MCC as the starting material. As shown in Figure 2a, TDMS-Cl reacts with the hydroxyl groups on the cellulose chains to form covalent Si–O–C bonds. Combined with the subsequent quantitative analyses of DS by ^1^H NMR and XPS, the DS of the as-prepared TDMS-Cell was determined to be approximately 2.0, which is consistent with values reported in the literature [18]. The purified product was obtained as a white powder and exhibited hydrophobicity.

FTIR spectroscopy is a key technique for characterizing organic modifications, as it enables the identification of functional group changes upon modification and provides direct evidence for the successful grafting of target groups onto cellulose chains [23]. As shown in Figure 2b, MCC exhibits a strong hydroxyl (–OH) stretching vibration at 3450 cm^−1^ and a weak methylene (–CH_2_–)/methine (–CH–) stretching vibration at 2871 cm^−1^. In contrast, the –OH absorption in TDMS-Cell is significantly weakened, while the –CH_2_–/–CH– related absorptions are markedly enhanced, indicating that part of the hydroxyl groups of cellulose were substituted and hydrophobic alkyl moieties have been introduced. In addition, TDMS-Cell shows characteristic asymmetric stretching and bending vibrations of methyl (–CH_3_) groups at 2958 cm^−1^ and 1460 cm^−1^, respectively. New absorption bands appear at 777, 823, and 1250 cm^−1^, which are assigned to the characteristic vibrations of Si–O–C bond and are consistent with previous reports [24]. These spectral features confirm the successful introduction of silyl groups into the cellulose backbone.

Proton nuclear magnetic resonance (^1^H NMR) spectroscopy was further employed to elucidate the molecular structure of TDMS-Cell and quantify its DS. Figure 2c shows the ^1^H NMR spectrum of TDMS-Cell in deuterated chloroform (CDCl_3_). The strong signal at −0.3–0.4 ppm is assigned to H_11_ (bonded to Si in the TDMS group), while the intense resonance at 0.55–1.2 ppm corresponds to H_10_ in the TDMS side chains. The sharp weak peak at 1.35–1.75 ppm is assigned to H_9_ in the *t*-hexyl group, and the broad signals at 3.0–4.5 ppm arise from H_1_–H_8_ on the glucopyranose ring. These results indicate that the TDMS groups have been successfully and completely grafted onto cellulose, and the mutual interactions between them lead to the observed spectral features. This result agrees well with Heinze et al. [25]. Based on the integral area ratio between the characteristic protons of the TDMS side chain and the skeletal protons of the glucopyranose ring, the degree of substitution of TDMS-Cell was calculated to be approximately 2 using Equation (1).

Similarly, the chemical bonding environment of TDMS-Cell was further analyzed via the deconvoluted C 1s XPS spectrum (Figure 2d). The deconvoluted peaks at 288.10 eV and 286.90 eV correspond to C atoms in different chemical environments, C–O–Si (bonded to Si) and C–O–C (cellulose backbone), respectively, further confirming the successful introduction of silyl groups into cellulose molecules. XPS analysis revealed atomic contents (excluding H) of 75.65% C, 17.51% O, and 6.83% Si. The DS calculated from Equation (2) based on these elemental contents was also approximately 2.0, which is in good agreement with the ^1^H NMR result.

XRD was employed to analyze the changes in the crystalline structure of TDMS-Cell (Figure 2e). MCC exhibits overlapping diffraction peaks at 2θ = 14.7° and 16.4°, corresponding to the (1–10) and (110) planes, respectively, along with a strong (200) reflection at 2θ = 22.6° and a weak (040) reflection at 2θ = 34.5° [26]. In contrast, TDMS-Cell shows a new diffraction peak at 2θ = 7.96°, while the original (1–10) and (110) peaks shift to 2θ = 13.6° and 15.24°, respectively, and the (200) and (040) reflections nearly disappear. These changes indicate that the packing mode of cellulose chains is significantly altered upon TDMS grafting. In particular, the new peak at 2θ = 7.96° suggests that the bulky silane substituents enlarge the interchain spacing and substantially reconstruct the crystalline packing structure. These results are consistent with the FTIR, ^1^H NMR, and XPS analyses, further confirming that TDMS has been successfully grafted onto the cellulose backbone and has induced pronounced changes in its crystalline structure.

### 3.2. Characterization of Composite Films

**Figure 3:**
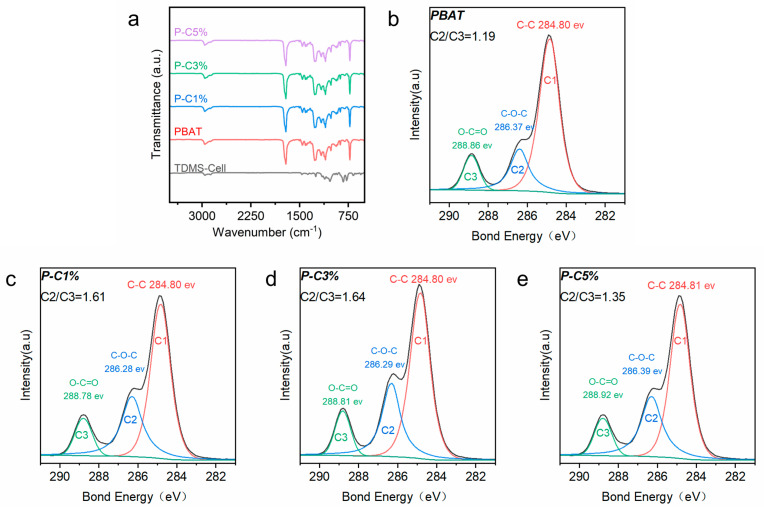
(**a**) FTIR spectrum of the P-C composite film. High-resolution C 1s XPS spectra: (**b**) PBAT, (**c**) P-C 1%, (**d**) P-C 3%, (**e**) P-C 5%.

Figure 3a shows the FTIR spectrum of the P-C composite films. Compared with neat PBAT, no new absorption bands appear in the P-C composite films, indicating that no new covalent bond structures detectable within the resolution of FTIR are formed. This suggests that no chemical bonds clearly identifiable by FTIR are generated between PBAT and TDMS-Cell, which is also commonly observed in other composite systems [27].

To further analyze the interfacial interactions between PBAT and TDMS-Cell, XPS was employed; Figure 3b–e show the high-resolution C 1s XPS spectra. The peaks at 286.3 eV (C2) and 288.8 eV (C3) correspond to two different carbon chemical environments in the PBAT backbone, namely C–O–C (ether) and O–C=O (ester/carboxyl), respectively. In the literature, the intensity ratio of the C2 and C3 peaks is often used as an indirect parameter to assess interfacial interaction changes in multiphase polyester systems [28].

The C2/C3 ratio of neat PBAT is 1.19 and increases to 1.61 upon the addition of 1 wt% TDMS-Cell, indicating that the incorporation of TDMS-Cell alters the distribution of interfacially related chemical environments and suggesting a significant change in the state of interfacial molecular interactions. We attribute this to the formation of abundant hydrogen bonds between the hydroxyl groups in the added TDMS-Cell and the PBAT matrix, which significantly alters the interfacial molecular interaction state. Similar increases in the C2/C3 ratio have also been reported in halloysite/PBAT and TPEE/graphene systems [29,30]. When the TDMS-Cell content is further increased to 5 wt%, the C2/C3 ratio decreases to 1.35, implying a reduction in the proportion of interfacially related chemical environments. Combined with subsequent morphological analysis, this decrease is attributed to the agglomeration of excessive TDMS-Cell, which reduces the effective interfacial interaction area.

**Figure 4:**
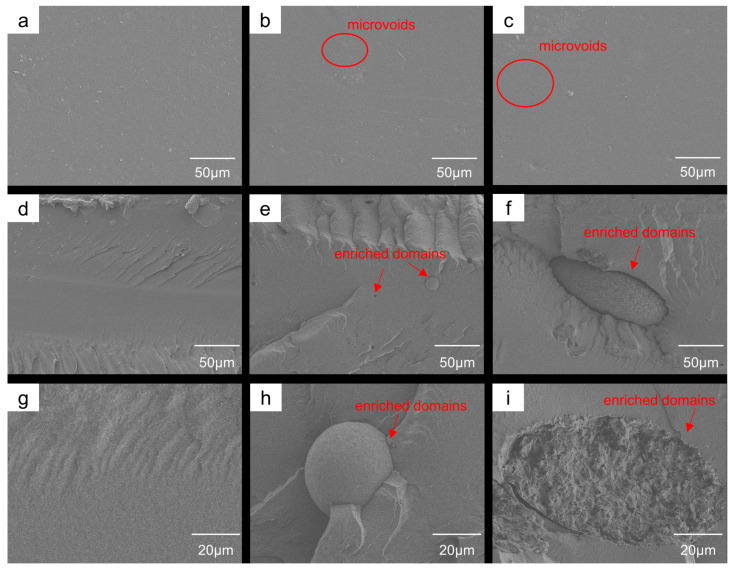
SEM images of the films: surface images at 500× magnification for (**a**) neat PBAT, (**b**) P-C1%, and (**c**) P-C5%; cross-section images at 400× magnification for (**d**) neat PBAT, (**e**) P-C1%, and (**f**) P-C5%; cross-section images at 1000× magnification for (**g**) neat PBAT, (**h**) P-C1%, and (**i**) P-C5%.

The interfacial structures of TDMS-Cell/PBAT composites at different loadings were further investigated by comparing the SEM morphologies of the film surfaces and cross-sections (Figure 4a–i and Supplementary Materials, Figure S1). The surface of the neat PBAT films is relatively smooth, with no obvious structural defects (Figure 4a). After the incorporation of TDMS-Cell, the surface morphology becomes rougher, with more localized microvoids observed (Figure 4b,c), indicating that TDMS-Cell modulates the surface morphology of the PBAT matrix. The cross-sections of neat PBAT films are relatively flat and exhibit typical ductile fracture features (Figure 4d). In the cross-section of the P-C1% sample, spherical TDMS-Cell enriched domains (~25 μm in diameter) are observed in the PBAT matrix (Figure 4e,h). When the TDMS-Cell content is further increased to 5%, large ellipsoidal agglomerates with sizes of about 80 μm, together with the appearance of local microcracks, are evident in the cross-section (Figure 4f,i).

These results indicate that although TDMS-Cell and PBAT are miscible in THF, a certain degree of phase separation still occurs during solvent evaporation and solidification, forming distinct TDMS-Cell-enriched domains whose size increases with increasing TDMS-Cell loading. The formation of such structural heterogeneities is generally detrimental to stress transfer and interfacial synergetic deformation; excessively large domains may reduce effective interfacial interactions [31]. This morphological evolution is consistent with the changes in interfacially related chemical environments revealed by XPS, collectively demonstrating the influence of TDMS-Cell loading on the interfacial structure and phase morphology of the composite system.

### 3.3. Crystallization and Thermal Stability Analysis of P-C Composite Films

**Figure 5:**
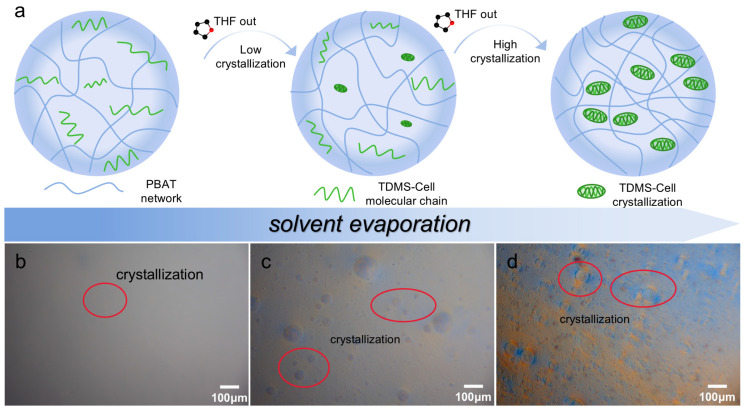
(**a**) Schematic illustration of TDMS-Cell precipitating and crystallizing during solvent evaporation, (**b**–**d**) OM images of P-C5% during solvent evaporation.

To elucidate the phase separation and crystallization behavior of TDMS-Cell in the PBAT matrix during solvent evaporation, schematic illustrations and optical microscopy were employed to track the morphological evolution of the system. Figure 5a schematically depicts the gradual precipitation and crystallization of TDMS-Cell from the PBAT/THF co-solvent system during solvent evaporation, while Figure 5b–d provide direct microscopic evidence of this evolution. Initially, PBAT and TDMS-Cell form a stable molecular-level dispersion with good compatibility in THF. As the solvent evaporates gradually, the polymer concentration increases, and TDMS-Cell precipitates and crystallizes. This process is likely associated with enhanced intermolecular interactions and an increased tendency toward phase separation [32]. Upon local enrichment, TDMS-Cell forms crystalline microdomains, similar to the crystal aggregation behavior reported in related crystallographic studies [33]. Driven by interfacial tension, these newly formed microdomains tend to reduce the interfacial free energy by decreasing their specific surface area and thus evolve into approximately spherical particles. Eventually, TDMS-Cell is dispersed in the PBAT matrix in the form of particulate domains. SEM observations further show that the size of the precipitated crystalline particles increases with increasing TDMS-Cell content, which is consistent with the enrichment-induced crystallization and particle growth process during solvent evaporation.

**Figure 6:**
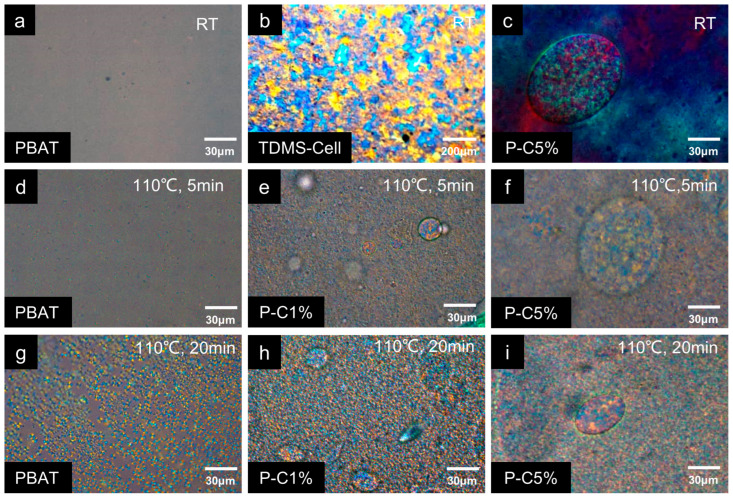
POM images: (**a**) neat PBAT, (**b**) TDMS-Cell, and (**c**) P-C5% at room temperature; (**d**) PBAT, (**e**) P-C1%, and (**f**) P-C5% after isothermal treatment at 110 °C for 5 min; (**g**) PBAT, (**h**) P-C1%, and (**i**) P-C5% after isothermal treatment at 110 °C for 20 min.

The influence of TDMS-Cell crystalline particles on the isothermal crystallization behavior of PBAT was further investigated by recording POM images of P-C composite films with different loadings during isothermal crystallization. As observed by POM, neat PBAT exhibits a semicrystalline state with relatively low crystallinity at RT, and no obvious crystallites are visible (Figure 6a). In contrast, the transparent TDMS-Cell films exhibit a highly crystalline structure, with large crystallites already present at room temperature (Figure 6b). In the P-C composite films, spherical particles formed by TDMS-Cell aggregation are clearly observed and exhibit a highly crystalline nature (Figure 6c). The size of these aggregated structures depends on the TDMS-Cell loading: the particle diameter in P-C1% is approximately 25 μm (Figure 6e), whereas the semi-major axis of the ellipsoidal particles in P-C5% can reach about 80 μm (Figure 6f), in good agreement with the SEM results showing an increase in enriched particle size with increasing TDMS-Cell content. After isothermal annealing at 110 °C, neat PBAT exhibits a typical spherulitic morphology. The number and size of the crystallites increase markedly with crystallization time, and the spherulite size reaches approximately 5–7 μm after 20 min of incubation (Figure 6g) [34]. Compared with neat PBAT, the P-C1% and P-C5% samples display much denser and smaller crystallites after isothermal treatment, consistent with the isothermal crystallization behavior reported by Li et al., [29]. These results indicate that TDMS-Cell incorporation accelerates PBAT crystallization while simultaneously inhibiting crystal growth. This behavior is attributed to the heterogeneous nucleation effect of TDMS-Cell on PBAT and the occupation of crystal growth space by pre-existing TDMS-Cell crystalline domains.

**Figure 7:**
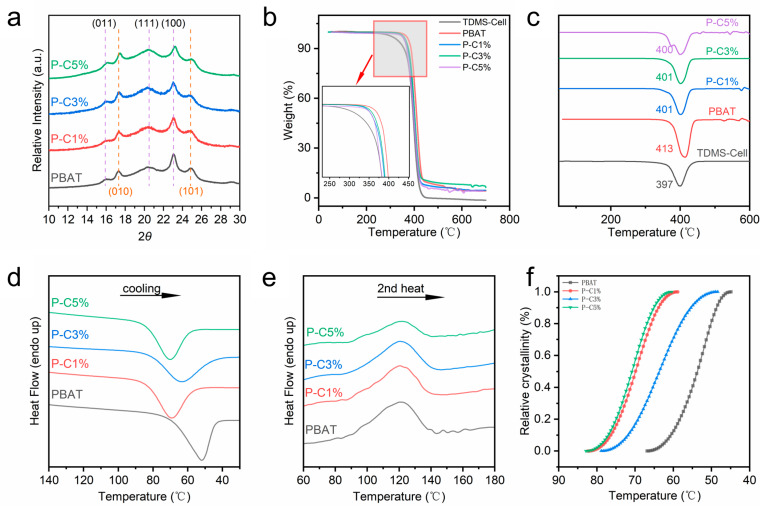
Crystallization and thermodynamic characterization of P-C composite films: (**a**) XRD patterns, (**b**) TG curves, (**c**) DTG curves; DSC curves at a cooling rate of 10 °C/min: (**d**) first cooling curves, (**e**) second heating curves, (**f**) relative crystallinity changes.

The XRD patterns (Figure 7a) show that all samples exhibit similar diffraction peak positions. Neat PBAT exhibits five characteristic reflections at 15.9°, 17.4°, 20.2°, 23.1°, and 24.6°, which are indexed to the (011), (010), (111), (100), and (101) planes, respectively, indicating an α-type triclinic packing structure [35]. In terms of peak shape, these characteristic reflections of neat PBAT are relatively weak and broadened, suggesting low crystallinity, which is consistent with the intrinsic semicrystalline nature of PBAT. A closer inspection of the peak positions reveals that, compared with neat PBAT, the diffraction peaks of the composite films shift toward higher angles with increasing TDMS-Cell loading, and this trend is particularly pronounced for the P-C5% sample. This shift indicates a reduction in interplanar spacing and the formation of a more compact crystal structure upon incorporation of TDMS-Cell [11].

To quantify the changes in crystallinity, the degree of crystallinity of different samples was calculated using the peak deconvolution method in combination with Equation (3). The crystallinity values of PBAT, P-C1%, P-C3%, and P-C5% are 26.1%, 27.3%, 19.42%, and 17.3%, respectively. With the incorporation of TDMS-Cell, the crystallinity of the composite films exhibits an overall trend of first increasing and then decreasing. This behavior indicates that when the TDMS-Cell loading exceeds a certain threshold, the crystalline fraction decreases and the internal structure becomes less ordered. Based on these results, a reasonable interpretation can be proposed: the introduction of a small amount of TDMS-Cell induces crystallization of the composites via a heterogeneous nucleation effect, promoting the formation of primary nuclei and slightly increasing the crystallinity of the P-C1% sample relative to neat PBAT. In contrast, at higher TDMS-Cell contents, excessive TDMS-Cell forms physical barriers that hinder further crystal growth, leading to reduced crystal size and, consequently, a pronounced decrease in the overall crystallinity. This trend is consistent with the POM observations and suggests that TDMS-Cell exerts a dual regulatory effect on the crystallization process—promoting nucleation at low loadings, while steric hindrance and inhibition of crystal growth dominate at high loadings.

TG analysis was performed, and the TG and DTG curves are shown in Figure 7b,c to evaluate the thermal stability of the composite films. The onset decomposition temperature and the temperature corresponding to the maximum mass loss rate are key parameters for assessing thermal stability (Supplementary Materials, Table S1). Neat PBAT exhibits an onset decomposition temperature of approximately 330 °C with a DTG peak at 413 °C, whereas neat TDMS-Cell shows an onset decomposition temperature of about 250 °C and a DTG peak at 397 °C. The DTG peak temperatures of the two materials are relatively close, while neat PBAT shows a slightly higher onset decomposition temperature, indicating certain similarities in their thermal degradation behavior.

After fabrication into P-C composite films, the overall onset decomposition temperature shifts slightly toward lower temperatures compared with neat PBAT, and the magnitude of this shift increases with increasing TDMS-Cell loading, yet it remains far higher than that of neat TDMS-Cell. When the TDMS-Cell content ranges from 1% to 3%, the DTG curves of the composite films exhibit a single peak, with the peak temperature lying between those of the two pure components and closer to that of neat TDMS-Cell. This behavior can be attributed to interfacial interactions and molecular chain entanglement between the two phases: PBAT chains inhibit and stabilize the thermal decomposition of TDMS-Cell, thereby suppressing its low-temperature degradation and shifting its decomposition toward higher temperatures. Meanwhile, the maximum decomposition temperature of PBAT shifts slightly toward lower temperatures under the influence of TDMS-Cell, and the overall mass loss behavior becomes more similar, indicating a certain synergistic effect between the two phases and good thermal stability of the P-C composite films at appropriate TDMS-Cell loadings. However, when the TDMS-Cell content reaches 5%, severe agglomeration leads to poor phase stability and the appearance of additional small decomposition peaks during thermal degradation.

DSC was employed to characterize the crystallization and melting behaviors of the TDMS-Cell/PBAT composite films [36]. DSC curves for the first cooling and second heating scans are shown in Figure 7d,e, and the corresponding parameters are summarized in Table 2. As shown in Figure 7d, during cooling, both neat PBAT and the P-C composite films exhibit distinct crystallization peaks with relatively narrow peak widths, and the peak crystallization temperature (T_c,p_) of neat PBAT is 52 °C. After incorporation of TDMS-Cell, the *T_c,p_* of all composite films shifts markedly to higher temperatures, whereas both the crystallization enthalpy (Δ*H_c_*) and the melting enthalpy (Δ*H_m_*) decrease significantly.

These results indicate that the nucleation efficiency and apparent crystallization ability of the P-C composite films are enhanced, while the overall degree of crystallization and the perfection of the crystal structure are reduced [37]. This suggests that TDMS-Cell exerts a dual regulatory effect on the crystallization behavior of PBAT: on the one hand, TDMS-Cell acts as a heterogeneous nucleating agent, restricting the random thermal motion of PBAT chains via intermolecular interactions and thereby increasing *Tc,p*; on the other hand, its own crystalline domains introduce significant steric hindrance, inhibiting crystal growth and leading to decreases in Δ*H_c_* and Δ*H_m_*. Similar behavior has been reported in other modified cellulose/PBAT systems [38,39]. In addition, the POM observations of decreased crystal size and increased crystal density are consistent with the reduced interplanar spacing and decreased crystallinity revealed by the XRD analysis.

To evaluate the influence of TDMS-Cell on the crystallization rate, the evolution of relative crystallinity under non-isothermal conditions was analyzed (Figure 7f). In non-isothermal crystallization kinetics, a shorter half-crystallization time (*t*_1_/_2_) corresponds to a faster crystallization rate. The *t*_1_/_2_ values were determined at cooling rates of 5 °C/min and 10 °C/min, respectively (Supplementary Materials, Table S2). The results show that, at both cooling rates, the *t*_1_/_2_ values of P-C1% and P-C5% are slightly shortened, whereas that of P-C3% is slightly prolonged. This non-monotonic trend can be attributed to the competition and partial compensation between the promoting and inhibiting effects of TDMS-Cell on PBAT crystallization, resulting in slight fluctuations in the overall crystallization rate. This behavior further reflects the complexity of the dual regulatory effect of TDMS-Cell on the crystallization process at low loadings.

### 3.4. Mechanical Properties and Hydrophobic Properties of P-C Composite Films

To investigate the effect of TDMS-Cell loading on the macroscopic properties, additional composite films with higher TDMS-Cell contents (10, 20, and 30 wt%) were prepared and denoted as P-C10%, P-C20%, and P-C30%, respectively. The effects of these high loadings on the mechanical properties and surface hydrophobicity of the composite films were systematically investigated.

**Figure 8:**
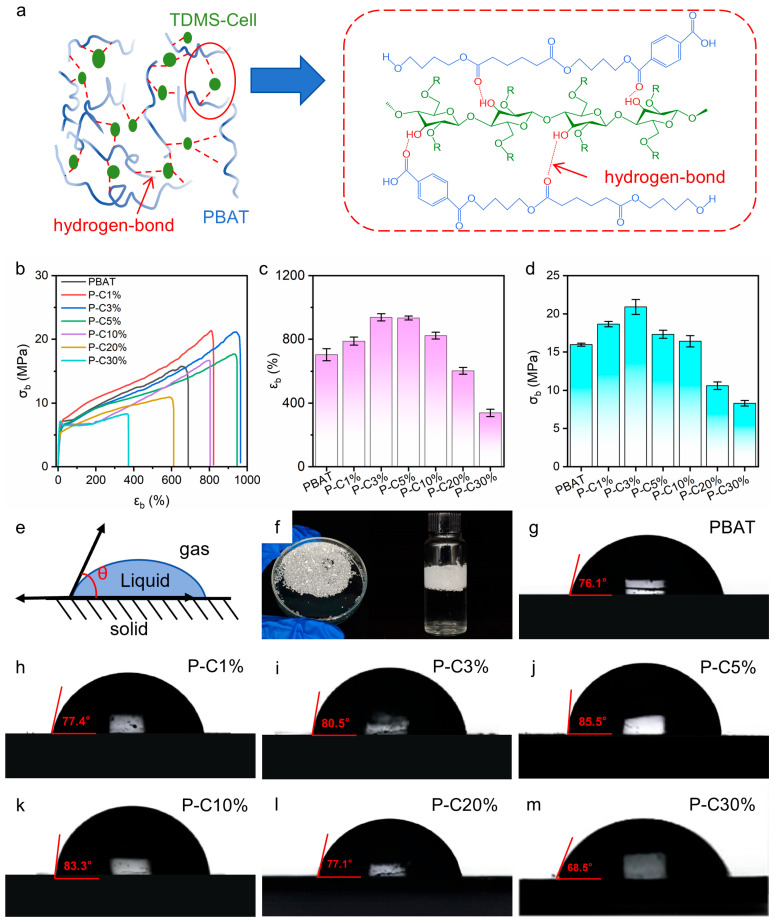
(**a**) Schematic diagram of interfacial interaction mechanism between PBAT and TDMS-Cell, (**b**) tensile stress–strain curves, (**c**) tensile strength (*σ_b_*), (**d**) elongation at break (*ε_b_*), (**e**) schematic diagram of contact angle measurement, (**f**) hydrophobicity of TDMS-Cell; contact angle images: (**g**) PBAT, (**h**) P-C1%, (**i**) P-C3%, (**j**) P-C5%, (**k**) P-C10%, (**l**) P-C20%, (**m**) P-C30%.

Tensile tests were carried out to quantitatively evaluate the mechanical performance of the composite films and assess the regulatory effect of TDMS-Cell incorporation on the mechanical behavior of the system (Figure 8b–d). Figure 8a schematically depicts the hydrogen-bonding interactions between TDMS-Cell aggregates and the PBAT matrix, which may influence the mechanical properties. For highly ductile polymer systems such as PBAT, the rapid stress rise at the initial stage of tensile deformation is attributed to the pre-yield elastic response, followed by an irreversible deformation stage dominated by plastic flow [15]. Figure S2 (Supplementary Materials) shows the elastic modulus variation in the elastic deformation stage. The elastic modulus of composite films changed slightly with 1–3 wt% TDMS-Cell loading compared to neat PBAT, but rose rapidly and exceeded that of neat PBAT at loadings over 10 wt%. This trend may be related to the poor interfacial compatibility between TDMS-Cell and PBAT at low loadings, which might facilitate molecular chain slip and thus lead to slight modulus variation. At high loadings, the formation of TDMS-Cell-enriched domains could construct a dominant network structure in the composites, which account for the sharp increase in elastic modulus [40,41].

Figure 8 shows the stress–strain curves of the composite films in the inelastic deformation stage; neat PBAT exhibits excellent ductility, with an elongation at break (*ε_b_*) of approximately 700% and a maximum tensile strength (*σ_b_*) of about 16 MPa. As shown in Figure 8c, the mechanical properties of the composite films are markedly improved when the TDMS-Cell loading is in the range of 3–5 wt%. Specifically, the maximum tensile strength (*σ_b_*) increases from approximately 16 MPa for neat PBAT to 21 MPa, corresponding to an improvement of 31.25%, which is superior to that of the PBAT/CNC system [38]. Meanwhile, the elongation at break (*ε_b_*) increases from about 700% to a maximum of 964%, representing a 37.7% increase. These enhancements are superior to those reported in PBAT/silanized cellulose composites [42]. This enhancement is consistent with the changes in interfacial interactions indicated by the XPS analysis and the phase morphology revealed by SEM. Combined with the above structural characterization results, it can be inferred that the interfacial synergetic deformation capacity of the composite system is enhanced within this loading range.

In sharp contrast, when the TDMS-Cell content exceeds 5 wt%, both the tensile strength and elongation at break of the composite films exhibit a pronounced decreasing trend. In conjunction with the SEM observations, this deterioration is attributed to significant agglomeration of excessive TDMS-Cell, which introduces large phase domains and structural defects into the system [43]. Such heterogeneities are unfavorable for efficient stress transfer and uniform stress distribution, thereby weakening the reinforcing effect and leading to a decline in mechanical performance. When the TDMS-Cell loading reaches 10 wt%, the tensile strength decreases to a level close to that of neat PBAT, indicating that under these conditions, the negative effects associated with TDMS-Cell agglomeration and interfacial discontinuity dominate and fully offset the reinforcement observed at low loadings. Within the present experimental system, the optimal TDMS-Cell loading for mechanical performance is therefore in the range of 3–5 wt%.

The water contact angle is an important parameter for evaluating the surface wettability of polymer films. The sessile drop method (gas–liquid–solid three-phase contact angle) was used to measure the water contact angles (Figure 8e), which reflect the variation in surface wetting behavior of the composite films after incorporation of TDMS-Cell [44]. Neat TDMS-Cell powder exhibits excellent hydrophobicity due to the grafting of silane-based hydrophobic groups, showing a non-wetting and non-absorbing state on the water surface (Figure 8f). Figure 8g–m present the water contact angles of PBAT and its composite films with different TDMS-Cell contents. The water contact angle of neat PBAT is 76.1°, indicating a weakly hydrophobic surface. Compared with neat PBAT, the water contact angle of the composite films first increases and then decreases with increasing TDMS-Cell loading. When the TDMS-Cell content reaches 5 wt%, the water contact angle increases to 85.5°, representing the maximum value. This result is similar to the hydrophobicity reported by Venkatesan et al. [45]. With further increasing TDMS-Cell loading to 20 wt%, the water contact angle decreases to 77.1°, which is slightly higher than that of neat PBAT but significantly lower than that at 5 wt% loading. When the TDMS-Cell loading reaches 30 wt%, the water contact angle further decreases to 68.5°, which is even lower than that of neat PBAT.

These results indicate that at low TDMS-Cell loadings, TDMS-Cell can be relatively uniformly distributed on the film surface, effectively masking hydrophilic hydroxyl groups and moderately reducing the surface energy, thereby increasing the water contact angle and enhancing surface hydrophobicity. At excessively high TDMS-Cell loadings, cellulose particles tend to agglomerate into large clusters, leading to reduced interfacial adhesion between TDMS-Cell and the PBAT matrix, as well as the formation of pores and microcracks. Such defects weaken the effective contribution of hydrophobic groups to surface wettability, ultimately resulting in a decrease in the overall hydrophobicity of the composite films and a corresponding reduction in the water contact angle, even below that of neat PBAT. Although the increase in water contact angle relative to neat PBAT is moderate, the observed trend in water contact angle with TDMS-Cell loading demonstrates that TDMS-Cell incorporation provides an effective approach to tailoring the surface wettability of PBAT films. This indicates the as-prepared film also shows promising applications in coated paper packaging [45] and food packaging [4].

## 4. Conclusions

In summary, this work realized molecular-level characterization of TDMS-Cell; developed a method for preparing TDMS-Cell/PBAT composite films via homogeneous THF solution blending; and systematically investigated their structural characteristics, mechanical properties, surface wettability, and crystallization behavior. The results show that the incorporation of TDMS-Cell shifts the crystallization temperature of PBAT toward higher temperatures, while simultaneously decreasing the Δ*H_c_* and Δ*H_m_*. In contrast, the *t*_1_/_2_ exhibits only slight fluctuations, indicating that TDMS-Cell exerts competing effects on PBAT nucleation and crystal growth. Additional samples with high TDMS-Cell loadings were introduced to further evaluate the mechanical and hydrophobic properties. With increasing TDMS-Cell loading, both the tensile strength (*σ_b_*) and elongation at break (*ε_b_*) of the composite films increase initially and then decrease, and the overall mechanical performance reaches an optimum in the low-loading range of approximately 3–5 wt%. Meanwhile, the surface water contact angle of the composite films shows a similar non-monotonic trend with increasing TDMS-Cell content, indicating that the incorporation of silanized cellulose enables the tailoring of surface wettability for PBAT composite films.

Combined with the multiscale characterization results (XPS, SEM, DSC, POM, etc.), it can be concluded that although TDMS-Cell and PBAT have similar polarities and are miscible in the same organic solvent system, TDMS-Cell still tends to form aggregated crystalline domains during solvent evaporation and solidification. These aggregates introduce structural discontinuities and weaken the interfacial synergistic deformation capability at high TDMS-Cell loadings. This structural evolution is consistent with the observed deterioration of mechanical properties at high TDMS-Cell contents, indicating that the self-aggregation behavior of TDMS-Cell plays a critical role in governing the interfacial structure and macroscopic performance of the composite system.

## Figures and Tables

**Table 1:** Formulation of premixed solutions.

Sample Name	PBAT (g)	TDMS-Cell (g)
PBAT	2	0
P-C1%	2	0.02
P-C3%	2	0.06
P-C5%	2	0.1

**Table 2:** Enthalpy change data of composite films during the first cooling and second heating processes via DSC testing.

Sample	First Cooling		Second Heat	
	*T_c,p_* (°C)	Δ*H_c_* (J/g)	*T_m,p_* (°C)	Δ*H_m_* (J/g)
PBAT	52	18.35	121.04	13.94
P-C1%	69.4	16.8	120.92	13.26
P-C3%	63.7	17.39	120.64	12.57
P-C5%	70.4	15.63	121.71	6.94

## Data Availability

The original contributions presented in this study are included in this article/its Supplementary Materials. Further inquiries can be directed to the corresponding author.

## Supplementary Materials

The following supporting information can be downloaded at https://www.mdpi.com/article/10.3390/polym18070875/s1. Figure S1: SEM images of P-C3% film: (a) surface at 500× magnification, (b) cross-section at 400× magnification, (c) cross-section at 1000× magnification; Figure S2: Elastic Modulus of Composite Films; Table S1: Thermal Stability Parameters of Neat PBAT and PBAT/TDMS-Cell Composite Films; Table S2: Relevant Parameters at Different Cooling Rates During the DSC Cooling Process.
