# Supplementary material for: Homogeneously Blending PBAT with Silanized Cellulose for Composite Film: Characterization and Physicochemical Property
Source: Polymers (Basel). 2026 Apr 2;18(7):875. doi: 10.3390/polym18070875 (PMC13074739; doi:10.3390/polym18070875)
Supplement: Supplementary file 1 [file polymers-18-00875-s001.zip › polymers-4227518-supplementary.pdf]

Supplementary Material

Homogeneously Blending PBAT with Silanized Cellulose for Composite Film: Characterization and Physicochemical Property

Ce Zhao[1], Xinxin Yan[1], Zhou Zhou[1], Lukuan Guo[1], Shilong Yang[2], Zhen Chen[3], Fengwei Jia[4], Junlong Song[1], Jiaqi Guo[1*]*

[1] State Key Laboratory for Development and Utilization of Forest Food Resources and Jiangsu Provincial Key Lab of Sustainable Pulp and Paper Technology and Biomass Materials, Nanjing Forestry University, Nanjing, Jiangsu 210037, China

[2] Advanced Analysis and Testing Center, Nanjing Forestry University, Nanjing 210037, China

[3] State Key Laboratory for Development and Utilization of Forest Food Resources, Jiangsu Co-Innovation Center of Efficient Processing and Utilization of Forest Resources, Nanjing Forestry University, Nanjing, Jiangsu 210037, China; Department of Nuclear Medicine, The First Affiliated Hospital with Nanjing Medical University, Nanjing, Jiangsu 210029, China

[4] Shandong Henglian New Materials Co.,Ltd., Weifang, 261106, China

Corresponding author: yanxinx@njfu.edu.cn (X.Y.), jiaqi.guo@njfu.edu.cn (J.G.)

1. SEM images of P-C3%

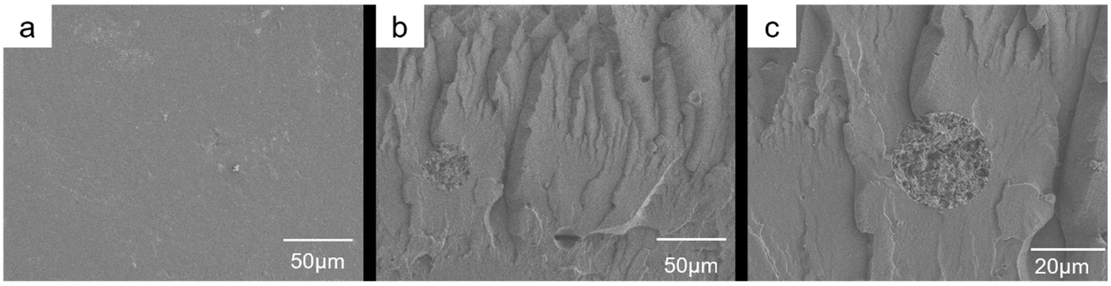

Figure S1. SEM images of P-C3% film: (a) surface at 500× magnification, (b) cross-section at 400× magnification, (c) cross-section at 1000× magnification.

2. Thermal Stability Parameters

Table S1 Thermal Stability Parameters of Neat PBAT and PBAT/TDMS-Cell Composite Films

Sample	T_{onset}/°C	$T_{10\%}$/°C	$T_{50\%}$/°C	$T_{90\%}$/°C	T_{max}/°C
TDMS-Cell	330.38	357.18	395.58	424.98	397.18
PBAT	376.73	386.73	410.33	442.43	413.73
P-C1%	359.94	372.74	400.14	426.04	401.74
P-C3%	364.94	375.94	402.04	464.44	401.24
P-C5%	360.19	368.69	398.09	424.99	399.99

T_{onset}: Initial decomposition temperature of the sample (defined as the temperature corresponding to 5% mass loss)

$T_{10\%}$: Temperature corresponding to 10% mass loss.

$T_{50\%}$: Temperature corresponding to 50% mass loss.

$T_{90\%}$: Temperature corresponding to 90% mass loss.

T_{max}: Maximum mass loss temperature from DTG curves

3.Calculation of Crystallization Parameters during the First Cooling Process in DSC Analysis

To further investigate the crystallization behavior of PBAT, the relative crystallinity (X_t) of PBAT with different TDMS-C contents was calculated. X_t was defined as the ratio of the partial curve area at a given crystallization temperature (T) to the total area of the crystallization curve from the onset crystallization temperature ($T_{c,o}$) to the end crystallization temperature ($T_{c,e}$). This ratio can be calculated using **Equation (S1)** as follows:

$$X_t = \frac{\int_{T_{c,o}}^{T} \frac{dH_C}{dT} dT}{\int_{T_{c,o}}^{T_{c,e}} \frac{dH_C}{dT} dT} \tag{S1}$$

The crystallization enthalpy (ΔH_c) refers to the total crystallization enthalpy released within an infinitesimal temperature interval dT. The half-crystallization time ($t_{1/2}$) is the relative crystallization time t required when the relative crystallinity (X_t) reaches 50%. The crystallization time and temperature were adjusted by changing φ, and the temperature T was converted to time t using **Equation (S2)**.

$$t = \frac{|T - T_{c,o}|}{\varphi} \tag{S2}$$

Table S2. Relevant Parameters at Different Cooling Rates During the DSC Cooling Process.

Sample	Cooling rate(φ)	$T_{c,o}$(℃)	$T_{c,p}$(℃)	$T_{c,e}$(℃)	$t_{1/2}$(min)	ΔH_C(J/g)
PBAT	5	76.0	64.2	56.0	2.02	17.95
	10	66.8	52	44.9	1.32	18.35
1%	5	86.9	76.7	68.2	1.95	14.34
	10	82.2	69.4	58.7	1.25	16.8
3%	5	83.6	72	57.9	2.4	14.95

Sample	Cooling rate(φ)	$T_{c,o}$(℃)	$T_{c,p}$(℃)	$T_{c,e}$(℃)	$t_{1/2}$(min)	ΔH_C(J/g)
	10	78.7	63.7	48.2	1.52	17.39
5%	5	88.1	78.4	70.7	1.9	13.26
	10	82.5	70.4	60.3	1.32	15.63

4. Elastic Modulus of Composite Films with Different TDMS-Cell Loadings

The tensile modulus (E) of the composite films was calculated from the stress-strain curves in the elastic deformation stage obtained via uniaxial tensile tests. Linear fitting was performed for the elastic deformation range (strain: 2–7%), with the correlation coefficient R^2>0.99 required to ensure strict compliance with Hooke's law. The tensile modulus was defined as the slope of the fitted straight line, and the **Equation (S3)** as follows:

$$E = \frac{\Delta\sigma}{\Delta\varepsilon} \quad (S3)$$

where Δσ is the tensile stress increment (MPa) within the elastic deformation range, and Δε is the corresponding engineering strain increment (dimensionless).

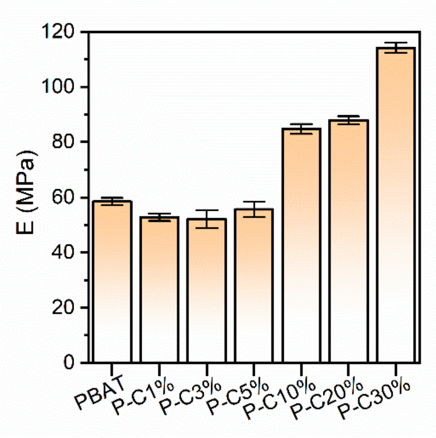

Figure S2. Elastic Modulus of Composite Films.
